# Gestational Diabetes—Screening, Prevalence and Postpartum Diabetes: Population‐Based Cohort Study

**DOI:** 10.1002/dmrr.70068

**Published:** 2025-07-17

**Authors:** Miri Lutski, Mor Saban, Debbie Novick, Amir Tirosh, Itamar Raz, Anat Tsur, Inbar Zucker

**Affiliations:** ^1^ Israel Center for Disease Control (ICDC) Ministry of Health Sheba Medical Center Ramat Gan Israel; ^2^ School of Public Health Gray Faculty of Medical & Health Sciences Tel Aviv University Tel Aviv Israel; ^3^ The Stanley Steyer School of Health Professions Faculty of Medicine Tel Aviv University Tel‐Aviv Israel; ^4^ Division of Endocrinology Diabetes and Metabolism Sheba Medical Center Ramat Gan Israel; ^5^ Gray Faculty of Medical & Health Sciences Tel Aviv University Tel Aviv Israel; ^6^ Diabetes Unit Department of Endocrinology and Metabolism Hadassah Medical Center Jerusalem Israel; ^7^ The Faculty of Medicine Hebrew University of Jerusalem Jerusalem Israel; ^8^ Department of Endocrinology and Metabolism Clalit Health Services Jerusalem Israel; ^9^ Medical Directorate Ministry of Health Jerusalem Israel

**Keywords:** cohort study, gestational diabetes, incidence, postpartum diabetes, registry, screening

## Abstract

**Aims:**

To evaluate GDM screening compliance and prevalence, and the association between gestational glucose intolerance and 5‐year postpartum diabetes mellitus (DM).

**Materials and Methods:**

We used population‐based data from three Israeli health maintenance organisations (HMOs), covering 75% of all births in 2016. GDM screening followed a two‐step approach: a 50‐g 1‐h oral glucose challenge test (OGCT), followed by a 100‐g 3‐h oral glucose tolerance test (OGTT) using Carpenter‐Coustan criteria. Data included age, socioeconomic status (SES), results of OGCT and OGTT tests, child birth weight, and gestational age. The dataset was linked to the Israeli National Diabetes Registry to identify postpartum DM. Logistic regression models estimated odds ratios (ORs) for GDM and postpartum DM, adjusting for maternal age, SES, ethnicity, and glucose tolerance status.

**Results:**

Among 128,454 women, 10% were unscreened. Of those screened, 23,451 underwent the full OGTT. GDM prevalence was 4.3%. Postpartum DM incidence was 8.6% in women with GDM, 3.1% with unknown GDM status, and 2.1% with impaired glucose tolerance (IGT) (defined as one abnormal value on the OGTT). Compared with normoglycemia, adjusted ORs for the 5‐year postpartum DM were 25.48 (95% CI: 21.80–29.79) for GDM, 10.04 (95% CI: 8.59–11.74) for unknown GDM status, 6.48 (95% CI: 5.07–8.28) for IGT, and 2.17 (95% CI: 1.63–2.88) for abnormal OGCT with normal OGTT. Older age, lower SES, and Arab or Bedouin ethnicity were linked to higher GDM and postpartum DM.

**Conclusions:**

Gestational glucose intolerance and screening gaps were strong predictors of postpartum DM. Age, SES, and ethnicity highlight the need for targeted efforts to reduce health disparities.

**Abbreviations**
CI: confidence interval
CVD: cardiovascular disease
DM: diabetes mellitus
GDM: gestational diabetes mellitus
HMO: health maintenance organisation
IGT: impaired glucose tolerance
INDR: Israeli national diabetes registry
OGCT: oral glucose challenge test
OGTT: oral glucose tolerance test
OR: odds ratio
PCOS: polycystic ovarian syndrome
SD: standard deviation
SES: socioeconomic status
T2D: type 2 diabetes

## 1 Introduction

Gestational diabetes mellitus (GDM) is a condition of glucose intolerance first recognized during pregnancy, which can be diagnosed at any stage of gestation, with early GDM (before 20 weeks) often associated with worse pregnancy outcomes compared with late‐onset GDM (diagnosed at 24–28 weeks) [1]. During pregnancy, rising insulin resistance peaks in late gestation, while *β*‐cell compensation varies [2]. In some women, inadequate *β*‐cell response leads to hyperglycemia, with early‐onset GDM linked to greater metabolic dysfunction [2]. The prevalence of GDM varies widely across regions and populations, ranging from approximately 7% in North America and Europe to over 27% in the Middle East and North Africa [3, 4, 5]. This variability is influenced by differences in diagnostic criteria, screening strategies, and population characteristics [1]. Additionally, global trends, including increasing rates of impaired glucose tolerance (IGT), obesity, and type 2 diabetes (T2D), have contributed to a rising prevalence of GDM over time [1]. Risk factors for GDM include ethnicity, maternal factors such as advanced age, high parity, and overweight, as well as a family history of DM, excessive weight gain during pregnancy, short stature, polycystic ovarian syndrome (PCOS), history of previous GDM, and multifoetal pregnancies [1].

Effective management of GDM is essential to normalise maternal blood glucose levels and prevent complications for both the mother and the foetus. Short‐term consequences for women with GDM include obstetric complications such as gestational hypertension, pre‐eclampsia, and cesarean section. Neonatal complications include large for gestational age (LGA), macrosomia, neonatal hypoglycemia, respiratory distress syndrome and birth trauma such as shoulder dystocia [1]. Women with a history of GDM have a significantly increased risk of recurrent GDM in subsequent pregnancies, with recurrence rates reaching up to 84% [6]. Additionally, they face a 20%–50% risk of developing T2D within 5–10 years postpartum. Offspring of mothers with GDM are also at heightened risk of early‐life obesity, insulin resistance, and long‐term cardiometabolic disorders [6].

Accurate data on GDM screening, prevalence, and complication rates are crucial for national healthcare planning. However, only a few countries, such as the United States [3], Finland [7], Germany [8], the United Kingdom [9], Norway [2], Denmark [10] and Sweden [11, 12] collect GDM‐related data through national medical birth registries, quality registries or surveillance systems that include antenatal care and pregnancy outcomes rather than dedicated GDM registries. Australia is the only country that collects GDM data through a dedicated registry; however, its coverage is limited to regional levels [13, 14].

GDM presents a growing global health concern, impacting both maternal and foetal health. Despite its significance, there is limited research on GDM prevalence and postpartum diabetes incidence in Israel, and national data on GDM screening compliance across different socioeconomic statuses (SES) and ethnic groups are lacking. Thus, this study aims to address these gaps by [1] determining compliance with GDM screening [2], estimating the prevalence rate of GDM, and the 5‐year incidence of postpartum DM, and [3] investigating the association between various degrees of gestational glucose intolerance and the incidence of postpartum DM, taking into account age, SES and ethnicity.

## 2 Materials and Methods

### 2.1 Subjects and Study Design

A population‐based retrospective cohort study was conducted, encompassing women who delivered in 2016, using data from three of the four health maintenance organisations (HMOs) in Israel: Clalit Health Services, the largest HMO; Maccabi Healthcare Service, the second largest HMO; and Leumit Health Services. In 2016, the combined number of births in these HMOs was 133,407, accounting for 75% of all births in Israel. For the current analysis, we excluded women with pre‐gestational DM (*n* = 711, 0.5%), those without a recorded week of birth (*n* = 3,625, 2.7%), and women with preterm births before week 30 (*n* = 617, 0.5%). Thus, a total of 128,454 women were included in the cohort (Figure S1).

### 2.2 GDM Screening

In Israel, universal GDM screening follows a two‐step process aligned with the American Diabetes Association guidelines [15]. Initially, a 50‐g oral glucose challenge test (OGCT) is conducted at 24–28 weeks. Women with a positive result (140 ≤ OGCT < 200 mg/dL; 7.8 ≤ OGCT < 11 mmol) undergo a 100‐g fasting oral glucose tolerance test (OGTT) [15]. Women at high risk, such as those with a history of GDM or obesity, usually perform the OGTT directly. All 128,454 women included in the cohort were categorised into three screening groups: (1) no screening—those who did not undergo any testing; (2) complete screening—those with a screening result of < 140 mg/dL (< 7.8 mmol/L) or ≥ 200 mg/dL (≥ 11.1 mmol/L), or a screening result of 140 ≤ OGCT < 200 mg/dL (7.8 ≤ OGCT < 11.1 mmol/L) who also underwent an OGTT, or those who underwent OGTT only; (3) Partial screening—those with a screening result of 140 ≤ OGCT < 200 mg/dL (7.8 ≤ OGCT < 11.1 mmol/L) who did not undergo an OGTT. In addition, women who did not undergo GDM screening or underwent partial screening were combined into a group categorised as having an unknown GDM status.

GDM was defined by 1‐h (h) post 50‐g OGCT plasma glucose ≥ 200 mg/dL (≥ 11.1 mmol/L), or based on the presence of two or more of the following values in the 100‐g OGTT: fasting serum glucose concentration exceeding 95 mg⁄dl (5.3 mmol⁄l); 1‐h serum glucose concentration exceeding 180 mg⁄d (10.0 mmol/L); 2‐h serum glucose concentration exceeding 155 mg⁄dl (8.6 mmol⁄l); and 3‐h serum glucose concentration exceeding 140 mg⁄dl (7.8 mmol⁄l) according to the Carpenter & Coustan criteria [16]. All other subjects in the complete screening group were categorised as non‐GDM. These non‐GDM women were further categorised into: 1. gestational IGT: one abnormal OGTT value; 2. abnormal OGCT with normal OGTT: OGCT ≥ 140 mg/dL (≥ 7.8 mmol/L) and < 200 mg/dL (< 11.1 mmol/L) with normal OGTT; 3. gestational normoglycemia: normal OGCT and normal OGTT values, as reported previously [17].

### 2.3 Diagnosis of Postpartum Diabetes

Diagnosis of postpartum diabetes was detected using the data from the Israeli National Diabetes Registry (INDR) between 2017 and 2021 years [18, 19]. The INDR was established in 2012 and is managed by the Israel Centre for Disease Control. A dataset of prevalent cases of diabetes from each of the four HMOs, which provide medical services to almost all Israeli permanent residents, is submitted annually to the INDR. The INDR consisted of all patients diagnosed with DM using the following entry criteria: patients with a diagnosis of diabetes (according to ICD‐9 9/ICD‐10 ‐ codes); test results of glycated haemoglobin (HbA1c) ≥ 6.5%; serum glucose concentrations ≥ 126 mg/dL (≥ 6.9 mmol/L) in at least two tests performed at least one month apart; purchases of diabetes medications.

### 2.4 Additional Covariates

Demographic data obtained included age at screening (categorised into five groups: 24 >, 25–29, 30–34, 35–39 and 40+). Residential SES was determined based on geographic statistical areas obtained from the HMOs using the residential address. HMOs utilise SES classifications provided by Points Location Intelligence, a company specialising in geographic and demographic data analysis [20]. The HMOs utilise SES classifications provided by Points Location Intelligence, a company specialising in geographic and demographic data analysis [20]. Points has developed a weighted socioeconomic index which is based on the classification defined by the Israeli Central Bureau of Statistics (CBS) data [21], combined with additional data collected by the company. The SES incorporates employment, housing conditions, education, financial status, and consumption patterns. Each geographic statistical area is ranked on a scale from 1 (lowest SES) to 10 (highest) [20]. The ethnic group was categorised as Jewish non‐Orthodox women, Orthodox Jewish women, Arab women (excluding Bedouin women), and Bedouin women, following the categorisation provided by residential locality and Points [20]. The data also included infant birth weight and gestational age at birth.

### 2.5 Data Analysis

The characteristics of the data were presented as counts with percentages and means ± standard deviations (SD). Chi‐square and analysis of variance tests were performed to identify significant differences in the characteristics and clinical outcomes among women who delivered in 2016 by the screening groups and by the gestational glucose intolerance categories.

Compliance with GDM screening tests was calculated as the proportion of pregnancies screened for GDM among the total number of live births in 2016. Age‐adjusted rates of women without any GDM screening were calculated for SES and ethnicity groups, using the 2016 Israeli general population as the standard. The 95% confidence intervals (CI) were computed using the normal distribution. The GDM rate per 100 persons was calculated by dividing the number of GDM cases in 2016 by the total of 128, 454 women who delivered that year, stratified by age.

The five‐year incidence rate of postpartum DM per 100 persons was calculated by dividing the number of incident postpartum DM cases during 2017–2021 by the total of 128,454 women who delivered in 2016 stratified by age and gestational glucose tolerance groups.

Logistic regression models were used to estimate odds ratios (OR) and 95% CI for GDM, adjusted for age and SES, and for age and ethnicity. Another logistic model was used to estimate OR and 95% CI for postpartum DM, adjusting for age, SES, ethnicity, and gestational glucose intolerance categories. Jewish non‐Orthodox women served as the reference group for ethnicity, high SES was the reference category for socioeconomic status, and normoglycemia was the reference group for gestational glucose intolerance categories. Additionally, we conducted a sensitivity analysis and performed logistic regressions to estimate ORs for postpartum DM, focussing exclusively on women diagnosed with GDM based on the 100‐g OGTT. These women were further categorised according to abnormal fasting glucose values versus abnormal post‐load values.

Statistical analysis was conducted using SAS Enterprise Guide 7.12 (SAS Institute Inc., Cary, North Carolina, USA).

## 3 Ethics

This study received approval from the Institutional Review Boards of Sheba Medical Centre Ethics Committee (Approval ID: SMC‐D‐0886‐23). Since the anonymised data were obtained from government computerised registers that serve regulatory purposes, an individual consent form was not required.

## 4 Results

The study included a total of 128,454 women who delivered at or after 30 weeks of gestation without known pre‐gestational DM. Compared to the excluded group (3625 women without a recorded week of birth and 617 with a preterm birth before week 30), the included women were older, had higher SES, and had a slightly higher prevalence of GDM, but similar five‐year postpartum DM incidence, as detailed in Table S1.

### 4.1 GDM Screening Compliance

Out of 128,454 included participants, 10.0% (*n* = 12,885) did not perform any GDM screening. Among the women who underwent a 50‐g OGCT and received results requiring further investigation (glucose levels ranging from 140 to 199 mg/dL, 7.8–11.0 mmol/L), 10% did not proceed to perform a 100‐g OGTT despite the guidelines (Figure S1). Characteristics of the 128,454 women were compared across the screening groups and all the characteristics were found to be statistically significant (Table 1). Women who did not undergo screening were younger, more likely to have low SES, and had a lower proportion of infants with birth weight ≥ 4000 g. The 5‐year postpartum DM incidence was highest among women with partial screening (4.1%), followed by those without screening (2.9%) and those with complete screening (0.8%) (Table 1). The age‐adjusted rates of pregnant women without any GDM screening were 16.0 (95% CI: 15.3–16.8), 10.9 (95% CI: 10.3–11.5), 7.4 (95% CI: 6.9–7.8), and 6.2 (95% CI: 5.3–7.1) for low, intermediate‐low, intermediate‐high, and high SES, respectively (Figure S2A). The age‐adjusted rates of pregnant women without any GDM screening were 24.0 (95% CI: 22.3–25.8), 16.7 (95% CI: 14.9–18.5), 11.5 (95% CI: 10.4–12.6) and 8.3 (95% CI: 8.0–8.6) for Bedouin women, Orthodox Jewish women, Arab women (excluding Bedouin), and Jewish non‐Orthodox women, respectively (Figure S2B).

**TABLE 1:** Characteristics and clinical outcomes of pregnant women who delivered during 2016 by screening groups.

		No screening *n* = 12,885 (10.0%)	Complete Screening *n* = 113,977 (88.8%)	Partial Screening *n* = 1592 (1.2%)	Total (*n* = 128,454)
Mean age* [years (± sd)]		29.6 ± 5.9	30.6 ± 5.5	31.3 ± 6.0	30.5 ± 5.6
Age groups at screening*, *n* (%)	≥ 24	2866 (22.2)	16,938 (14.9)	241 (15.2)	20,045 (15.6)
	25–29	3888 (30.2)	32,408 (28.4)	384 (24.1)	36,682 (28.6)
	30–34	3308 (25.7)	36,679 (32.2)	465 (29.2)	40,452 (31.5)
	35–39	2069 (16.1)	21,337 (18.7)	346 (21.7)	23,752 (18.5)
	40+	754 (5.9)	6615 (5.8)	156 (9.8)	7252 (5.9)
Socioeconomic status* ^,^ ^a^, *n* (%)	Low	5277 (45.6)	27,297 (25.1)	516 (35.1)	33,090 (27.2)
	Intermediate–low	3454 (29.8)	30,271 (27.8)	426 (28.9)	34,151 (28.0)
	Intermediate–high	2169 (18.7)	34,006 (31.3)	372 (25.3)	36,547 (30.0)
	High	679 (5.9)	17,154 (15.8)	158 (10.7)	17,991 (14.8)
Ethnicity* ^,^ ^b^, *n* (%)	Bedouin women	1687 (16.3)	4677 (4.7)	122 (9.6)	6486 (5.8)
	Arab women excluding Bedouin	1706 (16.5)	12,575 (12.6)	211 (16.6)	14,492 (13.0)
	Jewish non‐Orthodox women	6051 (58.5)	77,391 (77.7)	871 (68.7)	84,313 (75.8)
	Orthodox Jewish women	895 (8.7)	5007 (5.0)	64 (5.0)	5966 (5.4)
Gestation week of delivery*, *n* (%)	31	49 (0.4)	158 (0.1)	10 (0.6)	217 (0.2)
	32–36	813 (6.3)	5935 (5.2)	131 (8.2)	6879 (5.4)
	≤ 37	12,023 (93.3)	107,884 (94.7)	1451 (91.1)	121,358 (94.5)
Child weight* ^,^ ^c^, *n* (%)	< 2500	402 (3.3)	3114 (2.9)	56 (3.9)	3572 (2.9)
	2500–3499	8260 (68.7)	71,589 (66.4)	968 (66.7)	80,817 (66.6)
	3500–3999	2815 (23.4)	27,520 (25.5)	347 (23.9)	30,682 (25.3)
	4000+	545 (4.5)	5650 (5.2)	80 (5.5)	6275 (5.2)
5‐year postpartum DM*, *n* (%)	No	12,510 (97.1)	113,062 (99.2)	1526 (95.9)	127,098 (98.9)
	Yes	375 (2.9)	915 (0.8)	66 (4.1)	1356 (1.1)

^a^ *n* = 6675 SES missing.

^b^ *n* = 17,197 Mixed population without specific classification.

^c^ Child birth weight data were included only for births occurring ≥ 37 gestation weeks (*n* = 12 missing). Birth weight was categorised as follows: < 2500 g (small for gestational age), 2500–3499 g (normal birth weight), 3500–3999 g, and ≥ 4000 g (large for gestational age), *n* = 12 child weight missing.

* *p*‐value < 0.0001.

### 4.2 GDM Prevalence

**FIGURE 1:**
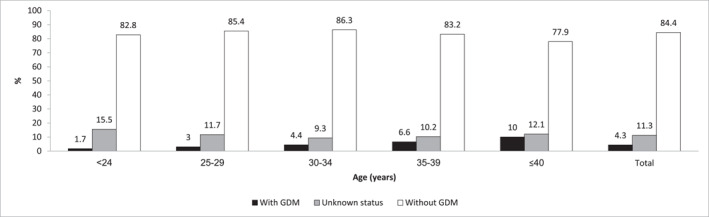
Distribution of GDM status by age groups. *Unknown status includes women who either did not undergo GDM screening or underwent partial screening.

Of the study population, 74.4% had gestational normoglycemia, 3.3% had an abnormal OGCT with normal OGTT, 6.4% had gestational IGT, and 4.3% had GDM. Compared to normoglycemic women, those with GDM were older, more likely to have higher SES, and had a significantly higher 5‐year incidence of postpartum DM (Table 2). GDM prevalence increased with age, from 1.7% in women < 24 years to 10.0% in those > 40 years (Figure 1).

**TABLE 2:** Characteristics and clinical outcomes of pregnant women who delivered during 2016 by gestational glucose tolerance groups.

		Normo‐glycaemia *n* = 95,569 (74.4%)	Abnormal GCT with normal OGTT *n* = 4229 (3.3%)	Gestational IGT *n* = 8659 (6.4%)	GDM *n* = 5520 (4.3%)	Unknown status^a^ *n* = 14,477 (11.3%)
Mean age* [years (± sd)]		30.3 ± 5.4	32.2 ± 5.6	31.5 ± 5.4	33.2 ± 5.6	29.7 ± 5.9
Age groups at screening*, *n* (%)	≥ 24	15,362 (16.1)	346 (8.2)	884 (10.2)	346 (6.3)	3107 (21.5)
	25–29	28,001 (29.3)	986 (23.3)	2332 (26.9)	1089 (19.7)	4272 (29.5)
	30–34	30,515 (31.9)	1444 (34.2)	2955 (34.1)	1765 (32.0)	3773 (26.1)
	35–39	16,904 (17.7)	1048 (24.8)	1815 (21.0)	1570 (28.4)	2415 (16.7)
	40+	4787 (5.1)	405 (9.6)	673 (7.8)	750 (13.6)	910 (6.3)
Socioeconomic status^b^ ^,^ *, *n* (%)	Low	23,561 (25.9)	845 (20.8)	1803 (21.7)	1088 (20.5)	5793 (44.4)
	Intermediate–low	25,315 (27.8)	1098 (27.0)	2268 (27.3)	1590 (30.0)	3880 (29.7)
	Intermediate–high	28,048 (30.8)	1380 (33.9)	2806 (33.8)	1772 (33.5)	2541 (19.5)
	High	14,130 (15.5)	746 (18.3)	1430 (17.2)	848 (16.0)	837 (6.4)
Ethnicity^c^ ^,^ *, *n* (%)	Bedouin women	4137 (5.0)	113 (3.0)	229 (3.0)	198 (4.1)	1809 (15.6)
	Arab women excluding Bedouin	10,687 (12.8)	412 (10.9)	950 (12.4)	526 (10.8)	1917 (16.5)
	Jewish non‐Orthodox women	64,157 (77.0)	3099 (82.2)	6169 (80.3)	3966 (81.2)	6922 (59.6)
	Orthodox Jewish women	4333 (5.2)	147 (3.9)	333 (4.3)	194 (4.0)	959 (8.3)
Gestation week of delivery*, *n* (%)	30–31	125 (0.1)	8 (0.2)	15 (0.2)	10 (0.2)	59 (0.4)
	32–36	4644 (4.9)	273 (6.5)	534 (6.2)	484 (8.8)	944 (6.5)
	≤ 37	90,800 (95.0)	3948 (93.4)	8110 (93.7)	5026 (91.1)	13,474 (93.1)
Child weight,^d^ ^,^ * grams, *n* (%)	< 2500	2608 (2.9)	219 (2.7)	110 (2.8)	177 (3.5)	458 (3.4)
	2500–3499	60,946 (67.1)	5183 (63.9)	2308 (58.5)	3152 (62.7)	9228 (68.5)
	3500–3999	22,732 (25.0)	2208 (27.2)	1201 (30.4)	1379 (27.4)	3162 (23.5)
	4000+	4504 (5.0)	500 (6.2)	329 (8.3)	317 (6.3)	625 (4.6)
5‐year postpartum DM*, *n* (%)	No	95,279 (99.7)	8596 (99.3)	4139 (98.9)	5048 (91.5)	14,036 (97.0)
	Yes	290 (0.3)	63 (0.7)	90 (2.1)	472 (8.6)	441 (3.0)

^a^ Included women with unknown status, who either did not undergo GDM screening or completed only partial screening.

^b^ *n* = 6675 SES missing.

^c^ *n* = 17,197 Mixed population without Ethnicity classification.

^d^ Child birth weight data were included only for births occurring ≥ 37 gestation weeks (*n* = 12 missing). Birth weight was categorised as follows: < 2500 g (small for gestational age), 2500–3499 g (normal birth weight), 3500–3999 g, and ≥ 4000 g (large for gestational age).

* *p*‐value < 0.0001.

In logistic regression adjusted for age and SES, older age was strongly associated with GDM, with ORs increasing from 1.68 in women aged 25–29 to 6.53 in those ≥ 40 years. Women with lower SES had higher odds of GDM compared with those with high SES. However, no significant association was found between ethnicity and GDM after adjusting for age (Table 3).

**TABLE 3:** Odds ratios (ORs) and 95% confidence intervals (CIs) for GDM and for 5‐year incidence of postpartum DM.

		GDM^b^		5‐year incidence of postpartum DM^b^	
		Model 1 OR (95% CI)	Model 2 OR (95% CI)	Model 1 OR (95% CI)	Model 2 OR (95% CI)
Age groups at screening	≥ 24	1 (reference)	1 (reference)	1 (reference)	1 (reference)
	25–29	1.68 (1.48–1.90)	1.68 (1.47–1.92)	1.62 (1.27–2.07)	1.60 (1.23–2.07)
	30–34	2.53 (2.24–2.86)	2.48 (2.19–2.82)	2.30 (1.81–2.92)	2.22 (1.72–2.86)
	35–39	4.02 (3.54–5.56)	3.91 (3.43–4.46)	3.08 (2.41–3.91)	2.90 (2.24–3.75)
	40+	6.53 (5.69–7.49)	6.35 (5.49–7.34)	3.94 (3.02–5.15)	3.74 (2.82–4.97)
Socioeconomic status	Low	1.22 (1.11–1.34)		2.21 (1.78–2.76)	
	Intermediate‐low	1.44 (1.32–1.57)		2.03 (1.64–2.52)	
	Intermediate‐high	1.22 (1.12–1.33)		1.57 (1.26–1.95)	
	High	1 (reference)		1 (reference)	
Ethnicity	Bedouin women		1.09 (0.94–1.27)		1.44 (1.15–1.79)
	Arab women excluding Bedouin		1.08 (0.98–1.19)		1.57 (1.33–1.85)
	Orthodox Jewish women		0.87 (0.75–1.01)		0.74 (0.54–1.00)
	Jewish non‐Orthodox women		1 (reference)		1 (reference)
Gestational glucose tolerance groups	GDM			25.48 (21.80–29.79)	25.19 (21.41–29.64)
	Unknown status^a^			10.04 (8.59–11.74)	10.76 (9.14–12.67)
	IGT			6.48 (5.07–8.28)	6.12 (4.72–7.93)
	Abnormal GCT with normal OGTT			2.17 (1.63–2.88)	2.14 (1.59–2.89)
	Normoglycemia			1 (reference)	1 (reference)
C‐statistic		0.637	0.633	0.837	0.834
Women diagnosed with GDM based on the 100‐g OGTT^c^	Abnormal fasting glucose value^d^			3.34 (2.59–4.59)	3.43 (2.67–4.41)
	Abnormal post‐load values^d^			1 (reference)	1 (reference)
C‐statistic				0.694	0.681

^a^ Unknown status includes women who either did not undergo GDM screening or underwent partial screening.

^b^ Model 1 adjusted for Age and Socioeconomic status; Model 2 adjusted for Age and Ethnicity.

^c^ Sensitivity analysis among women diagnosed with GDM based on the 100‐g OGTT (*n* = 4816), 2232 (46.3%) had an abnormal fasting glucose value. Of these 2232 women, 275 (12.3%) developed postpartum DM.

^d^ Abnormal fasting glucose value: ≥ 95 mg⁄dl (≥ 5.3 mmol⁄l); Abnormal post‐load values: 1‐h serum glucose ≥ 180 mg⁄dl (10.0 mmol/L); 2‐h serum glucose ≥ 155 mg⁄dl (≥ 8.6 mmol⁄l); and 3‐h serum glucose ≥ 140 mg⁄dl (≥ 7.8 mmol⁄l).

### 4.3 Postpartum DM

**FIGURE 2:**
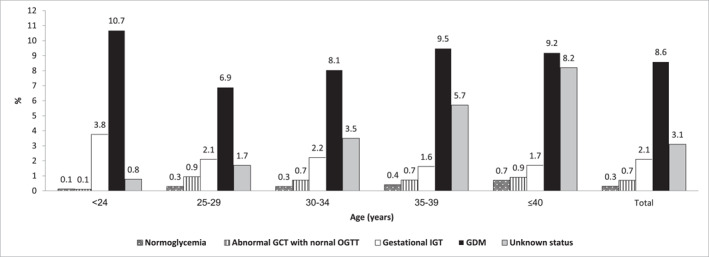
Distribution of 5‐year incidence rates of postpartum DM by gestational glucose tolerance groups* and age groups. *Unknown status includes women who either did not undergo GDM screening or underwent partial screening. Gestational IGT: one abnormal OGTT value; abnormal OGCT with normal OGTT: OGCT ≥ 140 mg/dL (≥ 7.8 mmol/L) and < 200 mg/dL (< 11.1 mmol/L) with normal OGTT; gestational normoglycemia: normal OGCT and normal OGTT values.

During five years of follow‐up, 1356 women (1.1%) developed postpartum DM. Incidence was highest among women with GDM (8.6%), followed by those with unknown GDM status (3.1%) and those with gestational IGT (2.1%) (Figure 2). Compared to normoglycemia, the adjusted ORs for postpartum DM were 25.48 for women with GDM, 10.04 for unknown GDM status, and 6.48 for IGT (Table 3). The risk of postpartum DM increased with older age and lower SES. In model 2, Bedouin and Arab (non‐Bedouin) women had significantly higher odds of postpartum DM compared with Jewish non‐Orthodox women (Table 3). Of the 4816 women diagnosed with GDM based on the 100‐g OGTT, 2232 (46.3%) had an abnormal fasting glucose value, among whom 275 (12.3%) developed postpartum DM. Among these women diagnosed with GDM based on the 100‐g OGTT, those with abnormal fasting glucose values had a 3.3‐fold higher risk of postpartum DM compared with those with normal fasting glucose levels (Table 3).

During the study period in 2016, the recommended postpartum screening was limited to HbA1c testing, without including the 75g oral glucose tolerance test. However, current guidelines, updated in May 2017, now recommend a 75g OGTT between 6 and 12 weeks postpartum. Additionally, our study findings indicate that approximately 81% of women with GDM did not undergo HbA1c testing within the first year postpartum (data not shown).

## 5 Discussion

### 5.1 Main Findings

Our main findings indicated that 10% of pregnant women did not undergo screening, and 10% of those with positive OGCT did not proceed to OGTT, resulting in 11.3% having unknown GDM status. A total of 4.3% women were diagnosed with GDM. Five‐year postpartum DM incidence rates were 8.6%, 3.1%, 2.1%, and 0.3% for women with GDM, incomplete screening, gestational IGT, and those with normoglycemia, respectively. Both older maternal age and lower SES were associated with GDM and postpartum DM. Additionally, being Bedouin or Arab women and having worse gestational glucose status or unknown GDM significantly influenced the risk of developing 5‐year postpartum DM.

### 5.2 Interpretation

Our findings are consistent with a previous Israeli study, which reported that 89.3% of pregnancies underwent GDM screening, showing minimal variation across different calendar years (1995–2009) [22]. This suggests that despite the availability of screening, there was no significant change in GDM screening compliance after 2009. In the Australian GDM register, estimated GDM screening rates were 96% in South Australia and 97% in Victoria (2012–2013) [13]. However, disparities in GDM screening rates are noted across different countries and populations, reflecting variations in healthcare policies, access to prenatal care, and cultural differences [4, 23]. For instance, despite New Zealand's universal screening recommendation for GDM, only half of the women attending antenatal care were screened, and ethnicity played a significant role in screening likelihood [24]. Similarly, in the US, racial and ethnic minority women, including Hispanic and Black women, may have different screening rates than White women, with some studies indicating higher screening likelihood due to clinical concerns [25].

The observed GDM prevalence of 4.3% in our study is lower than the global estimate of 14% reported by the 2021 IDF Diabetes Atlas, which is standardized based on the IADPSG criteria [4]. This relatively low prevalence raises questions given the universal screening coverage in Israel. Several factors may explain this discrepancy. The use of the more restrictive Carpenter‐Coustan criteria likely contributes to lower prevalence rates. Additionally, under‐screening among women from lower SES backgrounds, such as Bedouin, Arab, and Orthodox Jewish women, may lead to underdiagnosis and contribute to the seemingly low prevalence observed. Addressing these disparities is crucial for improving screening coverage and obtaining a more accurate estimate of GDM prevalence.

Consistent with prior studies, we found that older age and lower SES were associated with a higher risk of GDM. Women from the lowest SES quartile had a 22% higher risk of GDM compared with those from the highest quartile. Although ethnicity did not significantly impact GDM risk in our study, previous research, such as the Finnish Medical Birth Register, similarly identified lower SES as a significant risk factor for GDM [26].

Regarding 5‐year postpartum DM, adjusted ORs were highest in women with a history of GDM (24.48), followed by those with unknown GDM status (10.04), gestational IGT (6.48), and abnormal OGCT with normal OGTT (2.17). Our findings align with previous research indicating that women with GDM have a substantially higher risk of developing T2DM postpartum. Previous studies estimated the 10‐year risk of T2DM after GDM to range from 10.1% to 51.5%, depending on the severity of glucose intolerance [22]. Women with GDM have a 7.4‐fold higher risk for postpartum DM compared to normoglycemic women, while those with gestational IGT face a threefold increased risk [17, 27]. In the Medical Birth Registry of Norway, the risk of being dispensed medication for DM within the first postpartum years was estimated to be 41 times higher among women with GDM compared to those without [28]. Our findings align with previous research, including a Finnish cohort study, which found that GDM in women with normal weight was associated with a 10‐fold increased risk of DM, while the risk was 47‐fold in overweight women [26]. This highlights the significant long‐term metabolic risks of GDM, regardless of pre‐pregnancy weight, and emphasizes the importance of tailored postpartum follow‐up. However, the magnitude of the risk varies considerably across studies due to differences in clinical follow‐up routines, and in prevalence of risk factors for diabetes such as obesity and eating habits. These variations underscore the complexity of managing postpartum follow‐up and the need for standardized, evidence‐based guidelines to mitigate future morbidity.

### 5.3 Strengths and Limitations

This study utilises a comprehensive, nationally representative dataset with universal screening to assess GDM screening compliance, GDM prevalence, and the incidence of postpartum DM. The availability of OGTT values allowed for detailed categorisation of glucose tolerance, enhancing analytical precision and providing insight into varying degrees of gestational glucose intolerance. However, approximately 11% of pregnant women in our cohort did not participate in or complete routine prenatal screening, potentially leading to an underestimation of GDM prevalence. While a standardized national protocol for GDM screening is implemented across all HMOs in Israel—including the OGCT and OGTT—minor differences in laboratory calibration, equipment, or testing conditions may exist and could affect glucose measurements. These methodological variations should be considered when interpreting the findings [29]. A notable limitation is the absence of first‐trimester fasting glucose values and data on early pregnancy screening. In addition, the study included women insured by three of the four national HMOs, covering 75% of births in 2016, limiting full population coverage.

Data on the timing and frequency of postpartum glucose testing were unavailable, which may introduce bias in identifying postpartum DM cases. Notably, 81% of women with GDM did not undergo HbA1c testing within the first postpartum year, and we could not account for variations in testing time points. Since postpartum DM detection relies on appropriate follow‐up, this gap may lead to underestimation of true incidence. Furthermore, lifelong screening for prediabetes or type 2 DM is recommended for women with prior GDM, typically every 1–3 years based on individual risk. Our dataset lacked information on relevant maternal characteristics—such as pre‐pregnancy BMI, history of PCOS or fertility treatment, family history of DM, and pregnancy treatment modalities—factors strongly associated with GDM risk. We also could not account for potential confounders including dietary patterns, physical activity, and other lifestyle factors, which may vary by SES and ethnicity. The absence of such variables limits the precision of risk assessment and may result in residual confounding.

Lastly, the INDR does not distinguish between DM types diagnosed postpartum. Nonetheless, it is well established that most women developing DM after GDM are diagnosed with T2D [30].

### 5.4 Clinical Implications

GDM is a significant risk factor for the development of T2D and cardiovascular disease later in life [31, 32]. Therefore, improving adherence to postpartum interventions aimed at early detection and prevention of T2D is crucial. Despite the universal health insurance coverage in Israel, barriers related to cultural beliefs and lack of awareness—particularly among Bedouin, Arab, and Orthodox Jewish women—may reduce compliance with GDM screening and postpartum follow‐up. Healthcare systems should implement culturally sensitive education programs to promote awareness of the importance of GDM screening and postpartum surveillance [23, 32]. Importantly, the need for ongoing monitoring and promotion of a healthy lifestyle should not be limited to women diagnosed with GDM but should also extend to women who had isolated abnormal results on GDM screening tests, even if they did not meet diagnostic criteria for GDM. This group is also at increased risk and could benefit from preventive interventions. Finally, establishing clinical quality indicators for GDM screening, postpartum follow‐up, and long‐term metabolic monitoring could improve healthcare management and outcomes, ultimately reducing the burden of diabetes and its complications among women and their offspring.

## 6 Conclusions

Our study provides valuable insights into GDM screening patterns, GDM prevalence, and postpartum DM incidence, demonstrating differences in the risk of developing T2DM among various gestational glucose intolerance groups, including those with IGT and abnormal OGCT with normal OGTT. These findings emphasise the need for targeted interventions to reduce risk factors for future DM and improve postpartum follow‐up and screening practices to prevent future morbidity.

## Author Contributions

M.L., M.S., A.T., I.R., A.T. and I.Z. conceptualised this work and its design. M.L., M.S., and D.N. were in charge of the acquisition and initial analysis of the data. M.L. and M.S. further analysed the data and modelled it. M.L., M.S., A.T. and I.Z. interpreted the study results. All authors read and approved the final manuscript.

## Ethics Statement

All methods were performed in accordance with the ethical standards as laid down in the Declaration of Helsinki and its later amendments or comparable ethical standards. This study received approval from the Institutional Review Boards of Sheba Medical Centre Ethics Committee (Approval ID: SMC‐D‐0886‐23).

## Consent

Since the anonymised data were obtained from government computerised registers that serve regulatory purposes, an individual consent form was not required.

## Conflicts of Interest

The authors declare no conflicts of interest.

## Peer Review

The peer review history for this article is available at https://www.webofscience.com/api/gateway/wos/peer-review/10.1002/dmrr.70068.

## Supporting information

Figure S1

Figure S2

Table S1

## Data Availability

The dataset analysed during the current study is not publicly available due to privacy and ethical restrictions but is available from the corresponding author on reasonable request.
