# Supplementary material for: Gestational Diabetes—Screening, Prevalence and Postpartum Diabetes: Population‐Based Cohort Study
Source: Diabetes Metab Res Rev. 2025 Jul 17;41(5):e70068. doi: 10.1002/dmrr.70068 (PMC12269537; doi:10.1002/dmrr.70068)
Supplement: Supplementary file 1 — Figure S1 [file DMRR-41-e70068-s001.pdf]

Supplementary Figure 1: Cohort flowchart

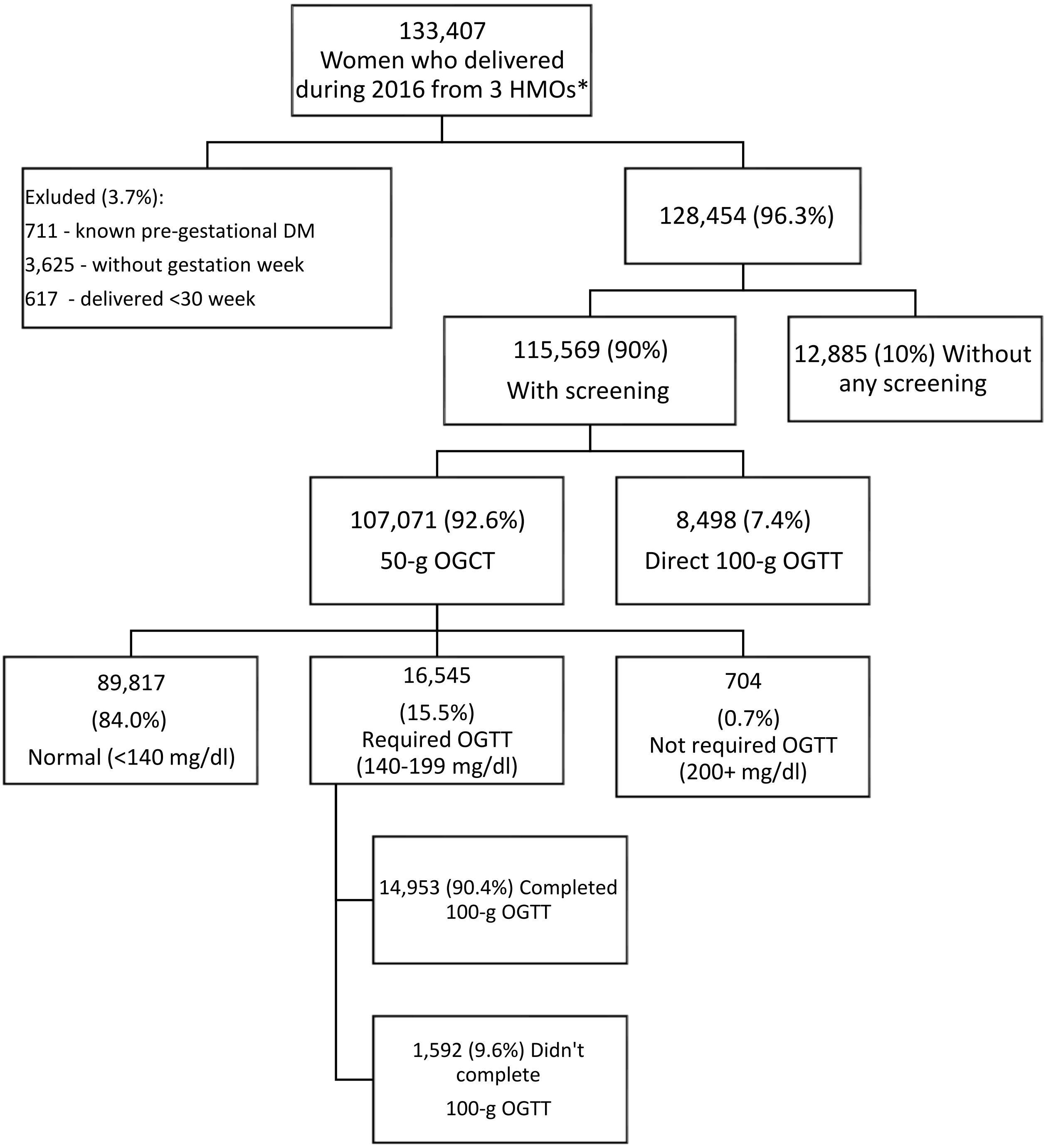

* In 2016, the combined number of births in these HMOs accounted for 75% of all births in Israel.
