# Supplementary material for: Gestational Diabetes—Screening, Prevalence and Postpartum Diabetes: Population‐Based Cohort Study
Source: Diabetes Metab Res Rev. 2025 Jul 17;41(5):e70068. doi: 10.1002/dmrr.70068 (PMC12269537; doi:10.1002/dmrr.70068)

Supplementary Figure 2: Age-adjusted rates of women without any GDM screening by SES and ethnicity groups

A. By SES groups

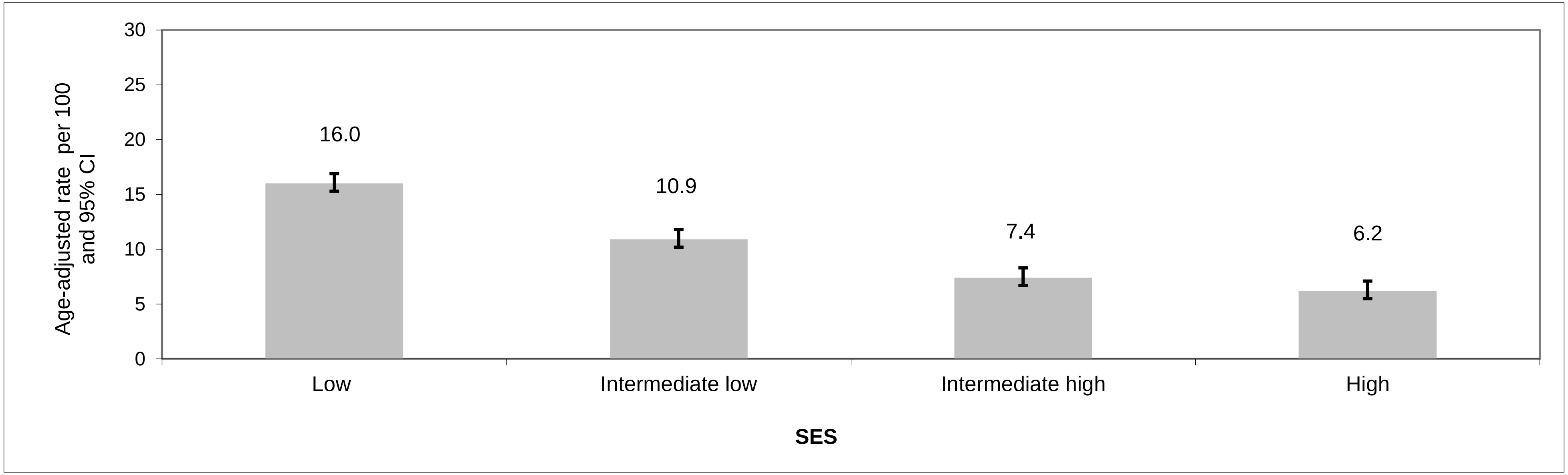

B. By ethnicity

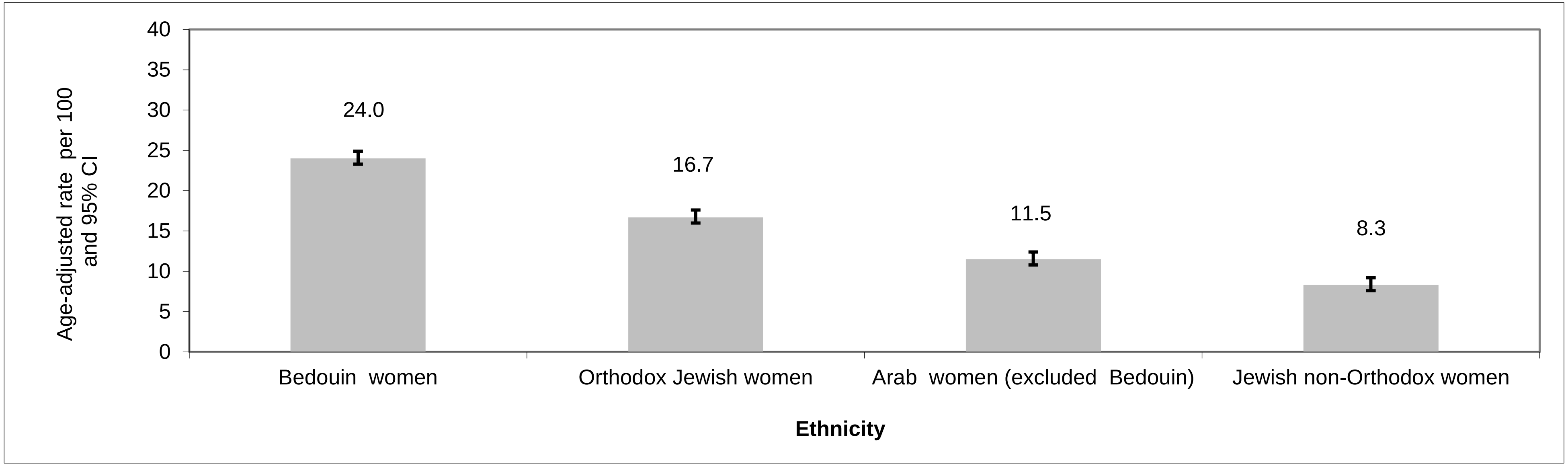

Supplement: Supplementary file 2 — Figure S2 [file DMRR-41-e70068-s003.pdf]
