# Supplementary material for: Gestational Diabetes—Screening, Prevalence and Postpartum Diabetes: Population‐Based Cohort Study
Source: Diabetes Metab Res Rev. 2025 Jul 17;41(5):e70068. doi: 10.1002/dmrr.70068 (PMC12269537; doi:10.1002/dmrr.70068)
Supplement: Supplementary file 3 — Table S1 [file DMRR-41-e70068-s002.docx]

**Supplementary Table S1:** The characteristics of the women who were included in the study compared to those who were excluded (3,625 without a recorded week of birth and 617 with a preterm birth before week 30).

| **P-value** | **Included**  **N=128,454** | **Excluded**  **N=4,242** |  |  |
| --- | --- | --- | --- | --- |
| <0.0001 | 30.5±5.6 | 29.1±6.2 |  | Mean age  [years (±sd)] |
| <0.0001 | 20,045 (15.6) | 1,183 (27.9) | 24≥ | Age groups*, n (%) |
|  | 36,682 (28.6) | 1,088 (25.7) | 25-29 |  |
|  | 40,452 (31.5) | 1,056 (24.9) | 30-34 |  |
|  | 23,752 (18.5) | 688 (16.2) | 35-39 |  |
|  | 7,252 (5.9) | 227 (5.4) | 40+ |  |
| <0.0001 | 33,090 (27.2) | 1,970 (53.6) | Low | Socioeconomic  Status †, n (%) |
|  | 34,151 (28.0) | 656 (17.9) | Intermediate‐low |  |
|  | 36,547 (30.0) | 716 (19.5) | Intermediate‐high |  |
|  | 17,991 (14.8) | 333 (9.1) | High |  |
| 0.0079 | 6,486 (5.8) | 149 (7.2) | Bedouin women | Ethnicity ‡, n (%) |
|  | 14,492 (13.0) | 286 (13.8) | Arab women excluded Bedouin |  |
|  | 84,313 (75.8) | 1,553 (74.8) | Jewish non-Orthodox women |  |
|  | 5,966 (5.4) | 89 (4.3) | Orthodox Jewish women |  |
| <0.0001 | 12,885 (10.0) | 1,228 (29.0) | No screening | Screening groups, n (%) |
|  | 1,592 (1.2) | 113 (2.7) | Partial screening |  |
|  | 113,977 (88.7) | 2,901 (68.4) | Complete screening |  |
| <0.0001 | 95,569 (97.4) | 2,548 (87.8) | Normoglycemia | Gestational glucose tolerance groups, n (%) |
|  | 8,659 (7.6) | 160 (5.5) | Abnormal GCT with normal OGTT |  |
|  | 4,229 (3.7) | 77 (2.7) | IGT |  |
|  | 5,520 (4.8) | 116 (4.0) | GDM |  |
| 0.974 | 127,775 (99.5) | 4,197 (98.9) | No | 5-years postpartum DM, n (%) |
|  | 1,356 (1.1) | 45 (1.1) | Yes |  |

† n=7,242 SES missing; ‡ n=19,362 mixed population without specific classification
